# Depression and sickness behavior are Janus-faced responses to shared inflammatory pathways

**DOI:** 10.1186/1741-7015-10-66

**Published:** 2012-06-29

**Authors:** Michael Maes, Michael Berk, Lisa Goehler, Cai Song, George Anderson, Piotr Gałecki, Brian Leonard

**Affiliations:** 1 Maes Clinics @ TRIA, Piyavate Hospital, 998 Rimklongsamsen Road, Bangkok 10310, Thailand; 2 School of Medicine, Deakin University, Kitchener House, Ryrie Street, Geelong, Victoria, 3220, Australia; 3 Orygen Youth Health Research Centre, Centre for Youth Mental Health, 35 Poplar Road, Parkville Victoria, 3052 Parkville, Australia; 4 The Mental Health Research Institute of Victoria, Australia Kenneth Myer Building, 30 Royal Parade, Parkville, Victoria, 3052, Australia; 5 Department of Psychiatry, Melbourne University, Level 1, North Block Main Building Royal Melbourne Hospital, Parkville Victoria, 3050, Australia; 6 Center for the Study of Complementary and Alternative Therapies, School of Nursing, University of Virginia, PO Box 800793, Charlottesville, VA 22908, USA; 7 Department of Psychology and Neurosciences, Dalhousie University, 1355 Oxford Streeet, Halifax B3H 4R2, Canada; 8 Department of Pharmacology, Chinese Academy Engineering Instit ute for the Development of Endangered Medicinal Resources in Southwest China 189 Changgang Road, Xing Ning District, Nanning, Guangxi Post Code 30023, P. R. China; 9 CRC, Rm 30, 57 Laurel street, Glasgow G11 7QT, Scotland, UK; 10 Department of Adult Psychiatry, Medical University of Łóź, Aleksandrowska 159, Łóź, 91-229, Poland; 11 Department of Pharmacology, Galway University, University Road, Galway, Co.Galway, Ireland

**Keywords:** depression, sickness behavior, inflammation, oxidative stress, cytokines

## Abstract

It is of considerable translational importance whether depression is a form or a consequence of sickness behavior. Sickness behavior is a behavioral complex induced by infections and immune trauma and mediated by pro-inflammatory cytokines. It is an adaptive response that enhances recovery by conserving energy to combat acute inflammation. There are considerable phenomenological similarities between sickness behavior and depression, for example, behavioral inhibition, anorexia and weight loss, and melancholic (anhedonia), physio-somatic (fatigue, hyperalgesia, malaise), anxiety and neurocognitive symptoms. In clinical depression, however, a transition occurs to sensitization of immuno-inflammatory pathways, progressive damage by oxidative and nitrosative stress to lipids, proteins, and DNA, and autoimmune responses directed against self-epitopes. The latter mechanisms are the substrate of a neuroprogressive process, whereby multiple depressive episodes cause neural tissue damage and consequent functional and cognitive sequelae. Thus, shared immuno-inflammatory pathways underpin the physiology of sickness behavior and the pathophysiology of clinical depression explaining their partially overlapping phenomenology. Inflammation may provoke a Janus-faced response with a good, acute side, generating protective inflammation through sickness behavior and a bad, chronic side, for example, clinical depression, a lifelong disorder with positive feedback loops between (neuro)inflammation and (neuro)degenerative processes following less well defined triggers.

## Introduction

The first inkling that there are phenomenological similarities between clinical depression and sickness behavior and that both conditions may share common pathways, that is, activation of the inflammatory responses system (IRS) was published in 1993 [1,2]. Sickness behavior is a behavioral complex that is typically induced by acute infections and tissue injury in many mammalian species. The characteristic behavioral pattern consists of malaise, hyperalgesia, pyrexia, listlessness and disinterest in social interactions with the environment, lethargy, behavioral inhibition, reduction of locomotor activity, exploration and grooming, reduction of reproductive performance, anhedonia, somnolence and sleepiness, anorexia and weight loss, failure to concentrate, and anxiety. There is evidence that sickness behavior is mediated through the effects of pro-inflammatory cytokines (PICs), such as IL-1, TNFα and IL-6 [3-10]. In this context, there is abundant evidence that clinical depression is an immuno-inflammatory disorder characterized by among other things increased levels of PICs and acute phase proteins, including C-reactive protein and haptoglobin [11-20].

Characteristic symptoms of major depression include anorexia, weight loss, fatigue, lethargy, sleep disorders, hyperalgesia, reduction of locomotor activity, and failure to concentrate (American Psychiatric Association). Moreover, 'vegetative symptoms' of depression, such as anorexia, weight loss, and psychomotor retardation, are significantly associated with inflammatory markers in clinical depression, such as increased levels of plasma haptoglobin, an acute phase protein, synthesis of which is induced by the three abovementioned PICs [1,2].

**Figure 1:**
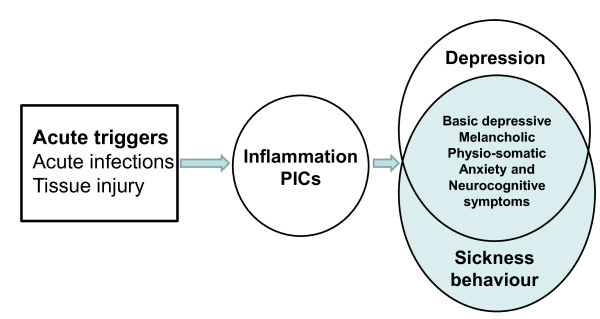
**Inflammation causes sickness and depression**. This Figure shows the theory that acute triggers cause inflammation and increased production of pro-inflammatory cytokines (PICs), which is associated with the onset of sickness behavior and clinical depression.

Thus, it may be concluded that there are striking behavioral and inflammatory similarities between both sickness behavior and clinical depression [1,2,11]. Therefore, some authors regard clinical depression as a form of sickness behavior and/or as a consequence of the sickness behavioral response [9]. Figure 1 shows the theory - principally derived from inflammatory translational models - that inflammatory triggers cause IRS activation and induce the production of PICs, which in turn may provoke sickness behavior and depression, thus explaining the phenomenological overlap between these conditions.

The main aims of this paper are to delineate: a) the symptomatic and behavioral similarities and dissimilarities between both clinical depression and sickness behavior; b) the staging of depression versus the course of sickness behavior; c) the shared immuno-inflammatory pathways that both underpin clinical depression and sickness behavior and may discriminate them; and d) the etiologic factors in both conditions.

### The nature of sickness behavior

Inflammatory inducers, for example acute viral and bacterial infections and inflammatory trauma, are detected by sentinels (for example, receptors on the innate immune system) and activate inflammatory mediators, for example, PICs which target the immune cells in the inflamed tissues [21]. Adaptive inflammatory responses are terminated once the triggers are eliminated and damaged tissues are repaired, a phenomenon known as resolution of inflammation. As we will explain in this section PIC-induced sickness behavior plays an important role in the resolution of inflammation and increased energy demands during inflammation.

### Sickness and energy consumption

Combating pathogen threats consumes large amounts of energy and, therefore, rations available energy [22]. PIC signals, for example, TNFα signals, modulate the balance between this increased energy demand and energy supply and control food intake, energy expenditure and substrate utilization [23,24]. PICs play a key role in this highly increased energy request characterized by a negative energy balance (increased lipolysis, loss of tissue proteins and lowered muscle protein synthesis, gluconeogenesis) and decreased voluntary energy utilization [22]. The central nervous system (CNS) receives neural and humoral signals about the peripheral inflammatory response through PIC-induced activation of afferent vagal signals, effects of TNFα at the sensory nuclei of the solitary tract, and all three PICs entering the brain though different pathways [22,25]. These PICs, in turn, will shut off energy-consuming processes, such as locomotor, neurocognitive and reproductive activity. Thus, metabolic energy is withdrawn from the brain and some peripheral organs and redirected to counteract the adverse effects of the invading pathogens. The energy saved by this process contributes to pyrexia and to the enhancement of the inflammatory state of immune cells [22]. Many of the sickness behavior symptoms, such as anergia, malaise, somnolence, psychomotor retardation, cognitive deficits and loss of libido serve to limit motor, sexual and brain activity and thereby direct metabolic energy to combating the primary infection [4,7,22,26]. Immune responses are highly calorie dependent and increase resting energy expenditure, while sickness behavior responses, for example, motor inhibition, may conserve critical energy [27].

### Sickness behavior and pyrexia

Mild to moderate pyrexia (in contrast to high fever) is a positive adaptive response as it strengthens the host defenses and resistance to infection, for example, by enhancing the phagocytosis and mobility of polymorphonuclear leukocytes and killing of bacteria and preventing viral replication [28-30]. In addition, PICs and cytokines, such as IFNs are more active during fever [31]. The relative balance of pyrogenic IL-1 and non-pyrogenic IL-18, and their endogenous inhibitory factors in different CNS sites, may contribute to the presence and degree of pyrexia [32]. In the past, fevers were even induced in patients to combat infections.

### Sickness behavior and anorexia and weight loss

Inflammation-induced anorexia is directly proportional to the magnitude of the insult and is also inversely related to body weight prior to the insult [33]. It is suggested that anorexia may limit the intake of iron, which otherwise would activate bacterial production [34]. Iron is one of the nutrients employed by bacteria for bacterial growth. This reasoning is, however, highly speculative as only less than 1% of dietary iron is absorbed and iron status changes slowly over time. A more plausible adaptive function is that anorexia through calorie restriction attenuates different intracellular signaling pathways leading to inflammation and even sickness behavior [35,36]. Thus, a two-week calorie restriction period significantly reduces inflammatory pathways, including IL-6 [37]. Calorie-restriction results in a dose-dependent suppression of lipopolysaccharide (LPS)-induced sickness behavior by inducing an anti-inflammatory state [36]. Motor inhibition and anorexia can lead to lean body mass loss, increased protein catabolism and loss of body protein and fat mass, which together may explain inflammation-induced weight loss and eventually cachexia. In the short-term, weight reduction, but not the composition of the diet, attenuates the inflammatory response, for example, by lowering inflammatory PICs, such as IL-6 and TNF [38-40] and increasing plasma levels of adiponectin, an adipose-derived anti-inflammatory protein [41].

**Figure 2:**
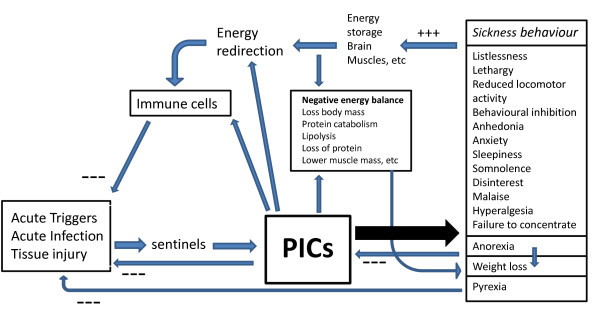
**This figure shows the functions of acute inflammation-induced sickness behavior**: **a**) energy saving by protecting the organism from the energy consuming effects of inflammation (through somnolence, lethargy, sleepiness, hyperalgesia, reduction of motor activity, exploration and grooming, cognitive deficits, loss of libido, anhedonia, disinterest in social interactions with the environment, and anxiety); **b**) anti-inflammatory effects (through anorexia, weight loss); and **c**) pathogen-directed effects (through pyrexia).

Figure 2 shows the different functions of acute inflammation-induced sickness behavior. Thus, sickness behavior is a short-term response to acute inflammatory triggers and is an adaptive motivational state induced to cope with these triggers and the consequent negative energy balance [4,7]. This homeostatic adaptive behavioral response is conserved across species through evolution and occurs in a predictable form cross-culturally [26].

### Symptomatic/behavioral similarities and dissimilarities between depression and sickness behavior

The Diagnostic and Statistical Manual of Mental Disorders (DSM IV-TR, American Psychiatric Association), one of the most commonly used diagnostic systems for psychiatric disorders, considers that the diagnosis of clinical depression can be made when at least five out of nine basic symptoms sare present for at least two weeks. Table 1 lists these nine basic symptoms and also three partially overlapping symptom dimensions that may superimpose on these basic symptoms, that is: 1) melancholic; 2) anxiety; and 3) physio-somatic dimensions [15]. It should be stressed that multivariate statistical analyses (pattern recognition methods) have confirmed and validated the basic symptoms of clinical depression and also the three symptom dimensions [42-44]. These three dimensions delineate specific depressive subgroups, for example, depression with melancholic, anxiety or physio-somatic features, which were externally validated by biomarkers.

**Table 1:** Characteristics of depression and sickness behavior.

	Clinical depression	Sickness behavior
Basic symptoms	Depressed mood most of the day Decreased interest or pleasure in almost all activities Anorexia, and/or significant weight loss or weight gain Insomnia or hypersomnia Psychomotor agitation or psychomotor retardation Fatigue or loss of energy Decreased ability to think or concentrate	-- Disinterest in social interactions Anorexia and weight loss; no weight gain Sleepiness Reduced locomotor activity; no agitation Lethargy Failure to concentrate
Existential symptoms	Feelings of worthlessness or guilt Suicidal ideation or behavior	-- --
Melancholic dimension	A distinct quality of depressed mood (anhedonia) Non-reactivity Diurnal variation Early morning awakening Psychomotor retardation Excessive weight loss	Reduced intake of sweetened milk (anhedonia) Behavioral inhibition -- -- Reduction of locomotor activity and exploration Important weight loss
Anxiety dimension	Tension; physiological behavior; respiratory symptoms; genito-urinary symptoms; autonomic symptoms; anxious behavior at interview (general)	Anxiety
Physio-somatic dimension	Flu-like malaise; aches and pain; muscle tension (in some of the patients)	Malaise and hyperalgesia (key symptoms of sickness)
Pyrexia	Slightly increased body temperature	Pyrexia
Onset Course	Insiduous Waxing and waning or relapsing-remitting Chronic Sensitization of episodes Seasonal variation (Hypo)manic episodes	Acute onset Acute adaptive response, maximal 19 to 43 days May be prolonged, but then is maladaptive -- -- --
Pathways	(Sub)chronic inflammation with increased PICs CMI activation Sensitization of inflammatory and CMI pathways Activation TRYCAT pathway O&NS Damage by O&NS Autoimmunity Neuroprogression	Acute inflammation with increased PICs Activated -- Maybe activated TRYCAT pathway Unknown but probably yes -- -- --
Triggers	Multiple, not well defined Psychosocial stressors, medical inflammatory illness, neuroinflammatory disorders, inflammatory conditions	Acute, highly defined Acute pathogens and tissue injury
	Episodes tend to become autonomous from trigger	Is always a response to a defined trigger
General	Inflammation-related chronic progressive disorder	Inflammation-induced adaptive behavioral response that is conserved through evolution
PICs' Janus-face	Bad 'chronic' side: a chronic disorder with positive feedback loops between (neuro)inflammation and (neuro)degenerative processes	Good 'acute' side: supports inflammation, redirects energy to immune cells, conserves energy and prevents negative energy balance, helps eradicating the trigger, and has anti-inflammatory effects

CMI, cell-mediated immune; O&NS, oxidative and nitrosative stress; PICs, pro-inflammatory cytokines; TRYCAT, tryptophan catabolites.

Table 1 compares the symptoms of clinical depression with the characteristics of sickness behavior. Basic symptoms/behaviors of clinical depression are also observed in sickness behavior, for example, anorexia, weight loss, reduction of locomotor activity and exploration, fatigue, sleepiness, and failure to concentrate. Moreover, symptoms belonging to the three dimensions, for example, melancholia (a failure to react to pleasurable stimuli, and excessive psychomotor retardation and weight loss) anxiety, and physio-somatic symptoms (malaise and hyperalgesia) also occur during sickness behavior. An apparent difference between depression and sickness behavior is pyrexia. There are, however, some reports that major depressed patients have increased body temperature as compared to normal volunteers, that is 98.38 ± 0.61 degrees F versus 98.13 ± 0.59 degrees F, respectively (mean ± SD) (that is, 36.88°C versus 36.74°C [45]. Increased body temperature in clinical depression is also shown by Szuba *et al. *[46]. However, there is by no means conclusive evidence of significant pyrexia in patients with clinical depression.

Obvious differences are suicidal ideation, feelings of guilt and worthlessness, symptoms indicating a distinctive existential state of depression [26]. In this context, it is important to note that Charlton proposed the malaise theory of depression, that is, malaise, the key symptom of sickness behavior, should be considered the core 'emotion' of depression. Indeed, a flu-like malaise or the subjective feeling of infection together with other physio-somatic symptoms is an important symptom dimension in clinical depression [44]. The malaise theory regards lowered mood and the more distinctive existential state of depression as a product of malaise. Thus, humans who suffer from sickness behavior and do not know they are ill, may interpret their lack of energy and neurocognitive disorders as a personal failure, causing feelings of guilt and unworthiness [26]. By inference, this theory considers depression not primary as an affective disorder but as a somatic disorder related to sickness behavior. Nevertheless, multivariate statistical analyses show that only some depressed patients suffer from physio-somatic symptoms, including malaise [44]. These findings contrast with the view that malaise is a core emotion of depression.

### Staging of depression versus the course of sickness behavior

Although some patients may suffer from one depressive episode only, longitudinal studies performed within the last two decades have shown that clinical depression is typically an episodic or lifelong disease [47-49]. Although depression was classically considered to be a self-limiting disorder with a short duration (six to nine months), the course of depression varies considerably. It may take the form of prolonged episodes (chronic depression), and depressive episodes may frequently return (recurrent depression). Moreover, up to 15% of depressed patients may develop treatment resistance, defined as a failure to respond to at least two adequate antidepressant trials [50]. A subgroup of depressed patients additionally suffer from hypomanic or manic episodes (bipolar depression), including abnormally elevated mood, energy and cognition; or from a mixed state with depressive and (hypo)manic symptoms occurring together. It should be underscored that the brain is one of the most metabolically active tissues, and energy and mood are intertwined. Depression is linked to reductions in brain energy generation and mania to increased energy expenditure [51], while sickness behavior is a behavioral response, which conserves energy.

Both unipolar and bipolar depression have characteristics of a progressive illness: they both follow stages, often commencing with mild or non-specific symptoms, progressing to prodromal features of subthreshold symptoms, then an acute episode, recurrence or a chronic form of persistent, unremitting illness [16,17,48]. An earlier onset of depression and increased length and number of episodes are associated with increased vulnerability to further relapses and a less favorable outcome [16,17,52]. This progressive course is associated with a functional deterioration, including neurocognitive decline [53,54]. This 'waxing and waning' pattern is similarly characteristic of autoimmune, progressive or degenerative disorders that combine an insidious onset with recurrent episodes or a chronic course and increased risk for functional deficits [11]. Moreover, there is evidence that sensitization offers a model for episode recurrence [55]. Sensitization indicates that repeated exposure to stressors, either psychological or organic, causes time-dependent, progressive increases in susceptibility to re-exposure to any of these stressors [56,57]. McEwen's allostatic load model has similarly been adapted to describe this progressive pattern [58]. Moreover, a seasonal variation is often found in unipolar and bipolar depression and depressive subgroups, including postpartum depression, with, for example, peak occurrences in spring [59,60]. There is also a seasonal variation in severity of depression and depressive symptoms, including suicide [61].

In contrast, sickness behavior is an acute, short-term state most often with an acute onset that is biologically appropriate to combat acute infections/trauma and thus enhances recovery. Evidently, (hypo)manic symptoms, the progressive deterioration over time, and seasonal variation are not characteristics of sickness behavior. Sickness behavior may be prolonged or inappropriately activated. Such a prolonged sickness behavior complex is likely to be dysfunctional [26] as is the case in more chronic infections and (auto)immune disorders. Motor and activity deficits are typically associated with sickness behavior [62,63] and form part of the fatigue - depression - sleep disorder cluster occurring during acute and chronic illnesses including heart disease, rheumatoid arthritis, and cancer [64]. In some individuals these symptoms can persist years after hospitalization, for example, post ICU and post-administration of radioactive substances. The question arises as to whether this condition should be regarded as sickness behavior given it is no longer beneficial? The following sections will address this question.

### Pathways underpinning clinical depression and sickness behavior

#### Inflammation and cell-mediated immunity

Studies in humans and rodents have defined PICs as central mediators of sickness behavior [5,6,65-67]. Inflammatory triggers induce 'neuraxes', that is, ascending neural pathways, which convey information about metabolic, gastro-intestinal and cardiovascular challenges to brain regions that mediate stress-related behaviors [68,69]. For example, infection and immune activation activate bottom-up, inflammatory pathways, mediated by PICs, from the periphery to the dorsal vagal complex, and ventrolateral medulla of the caudal medulla [9,70-72]. These inflammatory challenges to internal bodily functions are translated into the brain via viscerosensory pathways that subsequently drive sickness behavior, and depressive- and anxiety-like behaviors [72]. Moreover, PICs are actively transported to the brain by endothelial cell transporters or may diffuse through blood-brain-barrier deficient areas [25,72]. This may explain why systemic inflammation provokes neuroinflammation and microglial activation. For example, administration of LPS, a component of the bacterial wall of gram negative bacteria, causes neuroinflammation and microglial activation, characterized by increased levels of TNFα, which may remain elevated for months and are associated with the onset of sickness behavior [8]. Moreover, the same pathways may activate brain regions, for example, the bed nucleus of the stria terminalis, that mediate threat-related information and infection-induced anxiety [71,73-75]. Studies investigating the mechanisms of these behavioral deficits in acute illness models have shown that peripheral inflammation leads to activation of a 'danger pathway' that originates in the caudal brainstem and signals the presence of physiological stressors to regulatory brain regions including the hypothalamus. Activation of this danger pathway leads to the suppression of hypothalamic arousal systems, notably the tuberomammillary histaminergic system, associated with behavioral activity [68]. When activation of the danger pathway is prevented, motor aspects of sickness behavior are absent following acute inflammatory challenge [69]. Thus, reduction in behavior associated with sickness is concomitant with an active inhibition of arousal systems [76]. The danger pathway also provides input to other brainstem areas involved in the regulation of arousal including the serotonergic dorsal raphe nucleus [68,69], and in this way may serve as a link between peripheral inflammation and brain functions regulating and/or influenced by alterations in arousal states.

As reviewed previously, clinical depression and sickness behavior share the involvement of key pathways, the predominant one being the role of inflammation [15]. In clinical depression there is evidence of a chronic, low grade inflammatory process and cell-mediated immune (CMI) activation characterized by a T helper (Th1)-like response with activation of IFNγ-related pathways [77,78]. Recent meta-analyses and recent publications have confirmed increased levels of PICs in human depression particularly IL-6, TNFα, and IL-1, and CMI activation, evidenced by higher levels of neopterin and soluble IL-2 receptors (sIL-2Rs) [18-20,79,80]. Increased neopterin is a marker of increased IFNγ mediated macrophage activation. In humans, IFNα-based immunotherapy induces physio-somatic and depressive symptoms in many patients with hepatitis C virus. The onset of depressive symptoms during IFNα-based immunotherapy is strongly associated with induction of the cytokine network, including elevations in monocytic cytokines, Th-1-like and Th-2-like cytokines [18,81,82].

Elevated PICs and CMI cytokines are capable of producing depressive symptoms and administration of cytokines provides a robust experimental model of the acute phase of depression. Studies in the rodent [15] provide strong evidence that PICs, for example IL-1, IL-6, and TNFα, and CMI cytokines, for example IL-2 and IFNγ, all at around 50 μg/kg, may induce: a) sickness behavior; b) depressive-like symptoms (including loss of motivated behavior, reduction of social investigation, anorexia, weight loss, decreased spontaneous locomotor activity, increased locomotion, memory impairment, impaired spatial memory, and enhancement of the amnesic effect of scopolamine); c) melancholic symptoms (including anhedonia as indicated by reduced preference for a solution of sucrose or chocolate milk in comparison to water, and changes in the circadian clock through effects at the suprachiasmatic nucleus); d) anxiety (including anxiogenic effects in the elevated plus maze and elevation of conditioned fear memory); and e) physio-somatic symptoms (including fatigue, hyperalgesia, and autonomic symptoms). Administration of LPS elicits not only sickness behavior but also depressive- (suppression of social interaction and activity in the open-field test, food consumption and body weight, memory dysfunction), anxiety-like and physio-somatic behaviors [15]. Cytokines such as IL-1 not only have potent primary signaling effects, but also modulate many neurotransmitters intimately involved in mood regulation such as serotonin and noradrenaline [83]. Recently, the inflammatory processes were linked with the neurocircuitry hypothesis of depression [84]. Thus, PICs modulate cortical-striatal-limbic circuits, which process reward-based and affective information and are responsible for core depressive symptoms [84].

Certainly, it is difficult to determine whether the depressogenic and anxiogenic effects of LPS and other inflammatory triggers are genuine or related to sickness behavior [85]. Since cytokine- and LPS-based models are accompanied by depressive-like and sickness behaviors it was often difficult to delineate which of these models are characteristic for acute depressive-like behaviors or for sickness behavior. Nevertheless, some groups have apparently separated initial sickness behavior from depressive-like behaviors. Dissociation between LPS-induced depressive-like behaviors and sickness behavior could be established by testing mice at different time points following administration of LPS [86]. Lacosta et al. (1999) [87] showed that systemic administration of IL-2 to mice induces reductions in exploration and impaired performance in the Morris water-maze that are not related to sickness behavior. Administration of intracerebroventricular (icv) IL-1β induces a sickness behavioral response (as indicated by reduced locomotor activity, lethargy, and reduced body weight) and a stress- or anxiety-like response [88].

Activation of the IRS is controlled at different levels whereby regulatory mechanisms specifically target selected control points, such as the production of pro-inflammatory mediators or the effects of mediators on the target tissues [21,89]. This regulation of the IRS response is an adapted compartmentalized regulatory response characterized by reflex inhibition that tends to silence an overzealous IRS [90]. Examples of inflammatory reflex inhibition are inflammation-induced increases in IL-10, a negative immunoregulatory cytokine, and transforming growth factor (TGF)β, an antiproliferative factor [21]. PIC-induced changes in neural pathways also participate in inhibiting acute inflammation, for example, enhanced production of glucocorticoids and catecholamines, and activation of cholinergic pathways [91]. For example, counter regulatory mechanisms are initiated to limit an infectious (sepsis) and non-infectious systemic inflammatory response syndrome [90,92,93]. This counter anti-inflammatory response syndrome is an adaptive response of the immune status that dampens an overzealous inflammatory response caused by pathogens or immune trauma [90,92,93].

The existence of comparable counter regulatory processes, in particular reflex inhibition, has been described in clinical depression [11,78]: increased synthesis of the IL-1 receptor antagonist (IL-1RA), which inhibits the function of IL-1; PIC-induced activation of the cortisol-axis and the consequent immunosuppressive activities of glucocorticoids; increased IL-6 production, which is protective by increasing the production of IL-10, IL-1RA and glucocorticoids; increased IL-2R levels which induce a state of IL-2 starvation by binding their ligand and limiting the amount of IL-2 needed for immune cell proliferation; increased production of some acute phase proteins, for example, haptoglobin, which act as immunosuppressive factors; increased prostaglandin production, which may suppress lymphoproliferative responses; and lowered plasma tryptophan levels (see next section). These counter anti-inflammatory response syndrome mechanisms may explain why the immune-inflammatory response in clinical depression is accompanied by signs of immunosuppression, such as decreased *ex vivo *natural killer cell activity and mitogen-induced lymphoproliferative disorders [11]. In analogy with the term counter anti-inflammatory response syndrome, which is typically coined as a counter-regulatory response to sepsis/systemic inflammatory response syndrome, we would propose to label this compensatory reflex system as 'compensatory (anti)inflammatory reflex system' (CIRS).

Sickness behavior supports the protective inflammatory response (helps to eradicate the trigger and redirects energy to inflammatory cells), protects against possible detrimental effects of inflammation (for example, negative energy balance), while at the same time acting as an anti-inflammatory reflex (anti-inflammatory effects of calorie restriction and weight loss). Therefore, sickness behavior itself should be regarded as a CIRS response to acute inflammation. Thus, while clinical depression is accompanied by a CIRS that downregulates the immuno-inflammatory response, sickness behavior is part of a CIRS.

#### The TRYCAT (tryptophan catabolite) pathway

Recently, a new pathway associated with inflammation has been established in some individuals with depression, that is, activation of the tryptophan catabolite (TRYCAT) pathway [94-97]. The first and rate-limiting enzyme of this pathway is indoleamine 2,3-dioxygenase (IDO; EC 1.13.11.52) [98]. IDO is activated by IFNγ and by PICs, such as IL-1 and TNFα, thereby inducing the catabolism of tryptophan leading to tryptophan depletion and increased synthesis of TRYCATs, for example, kynurenine, kynurenic acid, xanthurenic acid, and quinolinic acid. IDO is expressed in many organs, for example, kidney, lung, spleen, and duodenum, immune cells, and the brain, for example, astroglia and microglia [99,100]. Lowered plasma tryptophan frequently occurs in clinical depression and is strongly associated with biomarkers of inflammation (acute phase reactants, increased cytokine levels) and CMI activation (increased serum neopterin and sIL-2Rs) [94-96]. During IFNα-based immunotherapy the onset of depressive symptoms is strongly associated with IDO activation, as assessed by means of the kynurenine/tryptophan ratio [101]. Likewise, in the postnatal period lowered tryptophan and increased IDO activity are related to anxiety and depressive symptoms [102]. Acute tryptophan depletion causes a robust increase in depressive symptoms in vulnerable individuals [103].

Recent studies have shown that IDO activation may separate sickness behavior and depressive-like behaviors in the rodent [104]. Thus, in Wild type (WT) mice, inoculation with bacille Calmette-Guérin (BCG), an attenuated form of Mycobacterium bovis, elicits IDO activation and consequent elevations in PICs and CMI-cytokines, such as IFNγ, IL-1β and TNFα [104]. Inoculation with BCG caused an initial acute episode of sickness behavior that was followed by a chronic state of depressive-like symptoms starting one week after BCG administration. Sickness behavior was equally induced in WT and IFNγR(-/-) mice, whereas IFNγ and TNFα together are necessary to cause IDO activation in microglia and consequent depressive-like behaviors. Moreover, IDO-deficient mice are resistant to the depressogenic effects of BCG, while they show a normal inflammatory response following BCG administration [104].

These TRYCAT data obtained in animal experiments are, however, difficult to extrapolate to clinical depression, because the clinical results are controversial. Thus, initial research showed no changes in urinary excretion of TRYCATs, such as xanthurenic acid and kynurenine, after tryptophan loading in depressed patients [105-108]. Nevertheless, higher xanthurenic excretion rates were related to anxiety and a lowered availability of plasma tryptophan [105,108]. The latter findings show that a 'TRYCAT shunt' through activation of IDO lowered plasma tryptophan in individuals with depression. In a recent study [109], lower levels of kynurenic acid and consequently a relatively increased kynurenine/kynurenic acid ratio were observed in depression. This ratio might be important for the pathophysiology of depression as kynurenine and some of its catabolites, for example, quinolinic acid, are depressogenic, anxiogenic, excitotoxic and neurotoxic, whereas kynurenic acid is neuroprotective [96]. One study shows increased TRYCATs in adolescents with melancholic depression [110] and another study increased quinolinic acid in microglia of depressed suicide victims [111]. Sublette *et al. *[112] detected increased plasma kynurenine levels in suicide attempters with major depression. Recent data shows that aberrations in the TRYCAT pathway, classically seen as a hallmark of depression, may be more germane to somatization, suggesting that the classically labeled psychosomatic symptoms of somatization may be more appropriately termed physio-somatic symptoms [113]. Thus, in a study comparing depression, comorbid depression + somatization, somatization alone, and controls, plasma tryptophan was even lower in somatization than in depression, while the kynurenine/kynurenic acid ratio and the kynurenine/tryptophan ratio were significantly higher in patients with somatization than depression [113]. Plasma tryptophan was negatively, and both ratios were positively, correlated with severity of physio-somatic symptoms. In summary, although in acute inflammatory states (for example, during IFNα-based immunotherapy) IDO activation is strongly related to the onset of depressive-like behaviors, this appears not to be the case in clinical depression since only some of those individuals, that is, those with physio-somatic symptoms or suicidal behavior, show relative increases in TRYCAT levels.

IDO activation has protective CIRS functions: IDO-induced reductions in plasma tryptophan and increased TRYCAT formation may attenuate the primary immuno-inflammatory response, for example, by attenuating T cell activation and proliferation [11,15]. This reflex inhibition could, therefore, be involved in spontaneous remissions of clinical depression, consistent with the observation that depression is sometimes a self-limiting disorder [96]. This CIRS mechanisms may also explain the contradictory findings on the TRYCAT pathway in acute depressive states (for example, IFNα-induced depression where there is a strong association between TRYCAT aberrations and the onset of depression) and major depression (DSM-IV-TR criteria), where these associations are much weaker probably because lowered tryptophan and increased TRYCATs have attenuated the initial immune-inflammatory response. Nevertheless, increased levels of TRYCATs, for example, quinolinic acid, in the anterior cingulate gyrus may be detrimental and play a role in clinical depression.

#### Detrimental immuno-inflammatory pathways that discriminate depression from sickness behavior

Although there is some evidence that shared immuno-inflammatory pathways may underpin sickness behavior and clinical depression, they have divergent effects in both conditions. Thus, the inflammatory response in sickness behavior has a beneficial CIRS effect on the organism, whereas inflammatory pathways and their sequelae have detrimental effects in depression and in particular in recurrent and chronic depression.

#### Transition to sensitization and autoimmunity

There is evidence that immuno-inflammatory responses are sensitized by recurrent depressive episodes. Thus, neopterin, a biomarker of CMI activation, is significantly increased in depressed patients who have experienced two or more depressive episodes than in patients who suffered only one depressive episode [18,114]. Likewise, plasma IL-1 and TNFα are significantly increased in depressed patients who suffered from three or more depressive episodes [18]. Women with a lifetime history of depression have increased inflammatory biomarkers, including IL-6 and sIL-1RA, in the early puerperium as compared to women who have never suffered from depression [115]. Increased C-reactive protein (CRP) levels in depressed men predict not only severity of the current depressive episode, but also recurrent depression [116]. It is known that PICs mediate central sensitization, for example, behavioral responses to maternal separation [117,118]. This suggests that recurrent depressive episodes amplify the pathophysiological responses of depressogenic cytokines, potentially enhancing inflammation-induced behavioral responses. Since it is known that increased numbers of episodes increase the risk of recurrence and treatment resistance [17], the findings suggest that sensitization of immuno-inflammatory pathways increases vulnerability to develop new depressive episodes.

Immuno-inflammatory mechanisms may also explain the high degree of anti-5-hydroxytryptamine (5-HT) antibody activity in clinical depression (54.1%), and in melancholia (82.9%) as compared to normal controls (5.7%) [119]. This autoimmune activity directed against 5-HT is significantly associated with biomarkers of inflammation (increased IL-1 and TNFα) and CMI activation (increased neopterin). In this respect, administration of pro-inflammatory and CMI-related stimuli, including LPS, IFNγ, and TNFα, reduces the survival of 5-HT neurons in the dorsal raphe nucleus, an effect that is not related to IDO activation [120]. The autoimmune activity directed against 5-HT is additionally associated with the number of previous depressive episodes, suggesting that exposure to previous depressive episodes increases anti-5-HT antibody activity, which, in turn, could confer increased risk to develop new depressive episodes.

Moreover, increased IL-6 and TNFα and lower serum zinc levels are associated with risk for depression and treatment-resistant depression [121-125]. This suggests that the immuno-inflammatory response in clinical depression may confer increased risk towards treatment resistance [126]. There is also evidence that immuno-inflammatory processes underpin the pathophysiology not only of unipolar and bipolar depression, but also mania [127,128]. Acute mania is accompanied by increased sIL-6R and sIL-2R levels, and increases in acute phase reactants [127,128], increased sCD4, sCD8, and sIL-1R antagonist levels [129], and elevated total immunoglobulin (Ig) G1, complement proteins C3, C6 and factor B [130]. Recently, it was argued that immuno-inflammatory processes play a role in the progressive shortening of the inter-episode interval with each recurrence when bipolar depression progresses [16,17]. All in all, the aforementioned findings suggest that sensitization (kindling) and progression of depression and bipolar disorder are, in part, caused by progressive inflammatory, CMI and autoimmune responses.

#### Transition to damage by O&NS processes

As with many inflammatory conditions, clinical depression is accompanied by activation of oxidative and nitrosative stress (O&NS) pathways [131]. The latter are likely amplified by reduced antioxidant levels, for example, coenzyme Q10, zinc and glutathione, and by the immuno-inflammatory responses in depression, perpetuating a vicious cycle between reduced antioxidants, inflammation and activated O&NS pathways [131]. There are no reports whether O&NS pathways play a role in adaptive sickness behavior. Nevertheless, these pathways have detrimental effects in clinical depression and additionally play a role in chronic depression. There is abundant evidence that depression is characterized not only by increased reactive oxygen and nitrogen species (ROS/RNS), but also by O&NS damage to lipids, proteins, DNA, and mitochondria [131]. In these processes O&NS pathways may alter the chemical structure of membrane fatty acids and functional proteins. When these modified fatty acids and proteins become immunogenic, an autoimmune response may be mounted directed against these 'neoepitopes' thereby further damaging the function or chemical structure of these epitopes [132,133]. Many depressed patients show increased IgM-mediated autoimmune responses directed against neoepitopes, such as the three anchorage molecules, palmitic and myristic acid and L-farnesyl S-cysteine, as well as acetylcholine, phosphatidylinositol, oleic acid, and NO-adducts, including NO-tryptophan and NO-tyrosine [133]. These autoimmune responses may interfere with cellular functions including intracellular signaling, apoptosis, and cellular differentiation. For example, the autoimmune reactions directed against the anchorage molecules may interfere with palmitoylation, myristoylation and farnesylation, and, therefore, with the binding and function of hundreds of proteins to the membrane [133]. Some of these IgM-mediated autoimmune responses are significantly higher in chronically depressed patients than in non-chronically depressed patients, suggesting that this O&NS damage may increase risk to develop chronic depression for example by activating neuroprogressive pathways [16,17,52,133]. These O&NS-related processes together with the effects of inflammatory mediators at 5-HT neurons [120] may explain the transition from inflammation and CMI activation to damage by O&NS and autoimmune reactions, which both may aggravate the preexisting inflammation and are involved in the process of chronic depression.

#### Transition to neuroprogression

Another immuno-inflammation-related pathway, which has detrimental effects in clinical depression and is not germane in sickness behavior, is neuroprogression, that is the stage related and potentially progressive process of neurodegeneration, reduced neurogenesis and neuronal plasticity, and apoptosis [15,17,123,126,134-137]. Many, but not all depressed individuals show features suggestive of a neuroprogressive illness. As discussed above, people who have a longer duration of illness and suffered from more frequent depressive episodes have a greater risk to develop subsequent relapses. Treatment response appears to reduce with more recurrent mood episodes [15,17,52]. Recurrent depressive episodes are correlated with increased cognitive disabilities, for example, decreased memory performance which is lowered by 2% to 3% following each depressive episode [138]. Depressive episodes are additionally associated with an increased risk of dementia [54]. Likewise, more depressive episodes are associated with underlying brain alterations, for example, reductions in volume of orbitofrontal and subgenual prefrontal cortex, hippocampus, and basal ganglia [52]. The duration of illness, for example, is negatively correlated with the volume of cerebral grey matter [139]. Meta-analysis showed that in patients with chronic depression (> 2 years) or recurrent depression hippocampal volume is significantly decreased and that the latter is related to the number of episodes [140,141]. In some studies, decreased hippocampal volumes in patients with recurrent depression are associated with neurocognitive decline [142]. Treatment resistance and the duration of illness are related to decreased right caudate and left putamen volume [52,143]. This effect may in part be related to a decrease in soma size of some cell types [144]. There is also evidence that in depression the abovementioned neuroprogression is at least partially caused by inflammatory and O&NS pathways [15,17,126]. PICs, such as IL-1 and TNFα, and CMI cytokines, for example, IFNγ and IL-2, TRYCATs, such as quinolinic acid, and damage by O&NS to structural fatty acids, anchorage molecules, functional proteins, DNA and mitochondria all may contribute to neuroprogression [15,17,126]. Further, both immune challenge and depression influence a constellation of brain regions that process viscerosensory and regulation of emotion in humans [145-147] and rodents [148]. These regions include parts of the medial prefrontal cortex (anterior cingulate) and basal forebrain (nucleus accumbens). Intriguingly, the CIRS seems to weaken with progression of the illness, and this failure to dampen inflammatory activation might play a role in the process of neuroprogression seen with multiple episodes [149].

All in all, the abovementioned findings show that clinical depression is accompanied by chronic inflammatory and O&NS responses and/or its sequelae, including sensitization, autoimmunity, damage by O&NS and neuroprogression. As a consequence, the inflammatory experiments described in the previous sections do not provide mechanistic explanations as to how PICs and CMI cytokines cause progressive clinical depression, as defined by its progressive course and progressive immuno-inflammatory pathophysiology. These experiments nevertheless support the view that PICs and CMI cytokines are associated with the onset of depressive-like behaviors and the physio-somatic, melancholic, and anxiety symptomatic clusters. However, most of these LPS- and cytokine-induced models did not separate sickness behavior from depression. In addition, these experiments are very limited because they select only one or two aspects of the symptom dimensions of depression, for example, reduced intake of sweetened milk as a model for anhedonia and thus melancholia [150]. More adequate animal models of clinical depression should be constructed to model not only the symptomatic dimensions, but also the typical course (self-limiting versus waxing and waning or progressive course) and the progressive pathophysiology (with sensitization and a transition to oxidative damage, autoimmunity and neuroprogression) of clinical depression.

#### Etiologic factors in depression and sickness behavior

Etiologically, sickness behavior is conceptualized as an acute phase of adaptive CIRS behavior in response to acute infections and inflammatory trauma [4,7,26]. When the resolution phase, however, is not induced, for example, when the IRS was unable to eradicate the pathogen, inflammation may persist despite the CIRS thus causing a chronic inflammatory state [21]. Chronic inflammation may result from a failure to eradicate the acute inflammatory trigger (for example, pyogenic bacteria), innately chronic irritants (for example, fungi, sarcoidosis), or autoimmune responses [151]. Acute and chronic inflammation are distinguished in terms of immune response patterns and time course. The time point of transition of acute inflammation to chronic inflammation is also related to the time when the energy stores become empty and this is estimated to be around 19 to 43 days depending on the nature of the energy store [152]. This transition is related to a number of inflammatory sequelae, including cachexia, insulin resistance, anemia, osteopenia and hypertension [152]. Sickness behavior thus plays a critical role in preventing the transition from acute to chronic inflammation following an acute trigger by compensating for the negative energy balance, redirecting energy to the activated immune cells, and so on [152]. Clinical depression, on the other hand, is accompanied by chronic inflammatory processes and is associated with less well defined trigger factors, as will be explained in this section.

In many chronic disorders, including degenerative (for example, diabetes, atherosclerosis) and neurodegenerative (for example, Parkinson's disorder) disorders the initiating trigger is not well defined, while the chronic state appears to consist of positive feedback loops connecting chronic inflammation and its pathophysiological process [21]. For example, Parkinson's disease (PD) is characterized by a vicious cycle between microglial activation and dopaminergic neuron degeneration, caused by detrimental effects of PICs, O&NS, and so on [25]. A similar pattern is observed in clinical depression: a chronic inflammatory process appears to be associated with transition to progressive autoimmune and neuroprogressive processes.

In contrast to sickness behavior, pathogens do not play a major role in clinical depression although some authors have attempted to make links between pathogens such as herpes simplex type 2 and toxoplasma gondii and psychopathology [153,154]. In a previous review, we concluded that there is no good quality evidence that acute infections and infections with Epstein-Barr Virus (EBV) may act as trigger factors associated with the onset of clinical depression [155]. Only some types of chronic infection are frequently associated with clinical depression, for example, HIV infection [156] and increased translocation of gram negative bacteria [157]. Some chronic infections, such as Lyme's disease can cause prominent neuropsychiatric sequelae [158,159]. Thus, while sickness behavior is an adaptive CIRS response to acute infections, only a few chronic pathogenic conditions appear to be associated with the onset of clinical depression. However, it cannot be excluded that reactivated dormant infected states and consequent infection-induced molecular pathways may be important in clinical depression. Viral infections of the brain, for example, cause neurologic and psychiatric dysfunction more often than appreciated [160].

Multiple trigger factors may provoke depressive behaviors, for example, psychosocial stressors, various medical disorders, and conditions as different as hemodialysis, IFNα-based immunotherapy, and the postnatal period [25,65,80]. Psychosocial stressors in humans may induce inflammatory, Th1-like and O&NS responses, including lipid peroxidation and DNA damage, while in the rodent different types of stressors induce peripheral and central activation of inflammatory, O&NS and neuroprogressive pathways [25,65,80,161]. For example, in the rodent, social defeat stress increases the reactivity of microglia to LPS, suggesting a role for social stress factors in the regulation of microglia responses [162]. Therefore, it may be concluded that psycho-traumatic and psychosocial stressors may cause depression and depressive-like behaviors through activation of immuno-inflammatory, O&NS and neuroprogressive pathways.

Many different medical disorders and conditions, which are associated with immuno-inflammatory and O&NS pathways, show a high comorbidity with depression: a) medical disorders, such as chronic obstructive pulmonary disease (COPD), cardiovascular disorder (CVD), chronic fatigue syndrome, obesity and the metabolic syndrome, rheumatoid arthritis (RA), systemic lupus erythematosus (SLE), inflammatory bowel disease (IBD), psoriasis, osteoporosis, and diabetes type 1 and 2; b) neurodegenerative or neuroinflammatory disorders, such as Alzheimer's (AD), PD and Huntington's disease, multiple sclerosis (MS) and stroke; and c) conditions, such as hemodialysis, IFNα-based immunotherapy, and the postnatal period [25,163]. We have argued that these disorders/conditions are all accompanied by activation of immuno-inflammatory and O&NS pathways and therefore may cause the basic immune/inflammatory state which may lead to comorbid depression [25]. Likewise, the same pathways could induce sickness behavior, which is experienced as feeling unwell, aches and pains, fatigue, and so on. However, while sickness behavior, per definition, is an adaptive response, comorbid depression worsens the aforementioned medical conditions [25]. Concomitant depression lowers the quality of life and increases disability and mortality in individuals with COPD, CVD, RA, SLE, IBD, psoriasis and diabetes type 1 and 2. Comorbid depression also contributes to lowered quality of life in individuals with MS, PD, AD, and stroke, negatively influences recovery from neurological defects, and predicts a higher morbidity and mortality in individuals with those neurological disorders. These negative effects of comorbid depression may be explained by increases in (neuro)inflammatory burden, including TRYCAT production, damage by O&NS, transition to autoimmunity and neuroprogression, which all together may drive the (neuro)inflammatory progression of the abovementioned medical conditions. During IFNα-based immunotherapy the incidence of depression was highest on the 12^th ^week of treatment, when more than 20% of patients with Hepatitis C Virus had moderate/severe depressive symptoms [164]. Physio-somatic symptoms, including fatigue, increased in the first week of treatment, and predicted cognitive-depressive symptoms some weeks to months later [165]. Thus, IFNα-based immunotherapy is probably one of the only trigger factors of depression that is characterized by transition from an acute inflammatory state (accompanied by sickness behavior) to a chronic inflammatory state (accompanied by depression).

Another factor that discriminates sickness behavior from depression is that the former is a response to a specific immune trigger, whereas the association between depressogenic triggers and depression is not always present. For example, as depressed people suffer from recurrent depressive episodes, it is less likely that stressful triggers are required to manifest depression. Following more than nine previous episodes, the trigger - depression association is muted and episodes appear autonomously, disconnected from triggers [166]. Also this effect may be explained by the knowledge that depression is a progressive disorder and that depressive episodes become sensitized [17].

The above suggests that chronic animal models such as the chronic mild stress and olfactory bulbectomized rat model are superior to the acute cytokine or LPS-induced models in that they: a) are less 'contaminated' with sickness behavior; and b) more accurately represent the psychosocial etiology, and/or (neuro)inflammatory and (neuro)progressive pathophysiology of clinical depression. Thus, the chronic mild stress model in the rodent shows that psychosocial triggers may cause depression-like behaviors in association with systemic and central inflammation and neuroprogression including decreased neurogenesis and neuronal cell damage [65]. The chronic mild stress model also mediates some of its effects from the differential regulation of the TRYCATs in different parts of the CNS [167]. A differential increase in quinolinic acid in the amygdala and striatum, with a trend increase in kynurenic acid in the frontal cortex following chronic mild stress would suggest an important role for variable TRYCAT pathway activation in mediating the changes associated with more psychosocial type stressors. Such differential TRYCAT pathway activation in different areas in the CNS may be relevant in differentiating sickness behaviors from clinical depression. Also, the olfactory bulbectomized rodent model of depression reflects the (neuro)inflammatory and neuroprogressive phenomena observed in clinical depression [66,67,168,169].

#### Is prolonged, exaggerated or maladaptive sickness behavior depression?

There is recent literature describing new concepts, such as prolonged, exaggerated and maladaptive sickness behavior [170,171]. In translational models, exaggerated inflammatory and neuro-immune responses are associated with so-called 'prolonge'" sickness behavior, including memory deficits. Such an exaggerated response is observed in aged as compared to adult mice [170]. This age-dependent prolonged sickness behavior is accompanied by oxidative damage to mitochondrial DNA (mtDNA) in microglia, increased intracellular ROS and activation of nuclear factor kappa B [170]. Subchronic administration of IL-1β significantly impairs spatial and learning memory, which is correlated with dysfunction of neurotrophins and their receptors [172]. Furthermore, the release of neurotransmitters, including acetylcholine, was significantly lower during memory retrieval. In hippocampal neurons, IL-1β administration significantly induced cell apoptosis, reduced αa-amino-3-hydroxy-5-methyl-4-isoxazolepropionic acid receptors, but increased N-methyl-D-aspartate receptor changes similar to those observed in AD [173]. It thus seems unlikely that depression is a maladaptive syndrome that results from prolonged 'sickness behavior' but rather the consequence of a chronic underlying immuno-inflammatory and degenerative process. These findings also show that there was no resolution to acutely triggered inflammation and consequently that transition towards chronic inflammatory pathology had occurred. By inference, this condition cannot be termed 'prolonged' sickness behavior, because the latter term indicates a short-term adaptive response to inflammatory trauma. Nevertheless, a valid conclusion is that shared pathways, for example, increased levels of PIC, explain the partially overlapping phenomenology of sickness behavior and clinical depression.

There is also a new hypothesis that associates depression as an evolutionary product of sickness behavior with protection from infection. As such depression is regarded as an evolutionary behavioral response that helps the immune system to fight pathogens and to avoid new pathogen exposure [26,174,175]. These hypotheses, however, did not take into account that clinical depression is not a simple behavioral response, but a progressive disorder driven by a cascading neurobiology leading to a progressive course and pathophysiology. Moreover, acute lethargy, hyperalgesia, loss of interest, anxiety, and anhedonia are beneficial behaviors, but when chronic the same symptoms are typically not beneficial, but pathological and maladaptive: they may further isolate the depressed patient from social contacts creating a state of demotivation and demoralization and negative anticipation of the future [26]. Chronic illness, furthermore, requires a person to use coping and adaptive strategies to integrate the consequences of a disorder. There is a wide diversity of coping styles and beliefs on the nature of depression, some of which are adaptive while others are maladaptive [176].

#### Antidepressive treatments

Antidepressants have significant immunoregulatory and immunosuppressive effects in normal volunteers and animal models. Tricyclic antidepressants (TCAs) and selective 5-HT reuptake inhibitors (SSRIs) attenuate the production of PICs, for example, IL-1β, TNFα and IL-6, and Th1-like cytokines, including IL-2 and IFNγ [177]. Most antidepressants, that is, TCAs, SSRIs, reversible inhibitors of monoamine oxidase A, 5-HT and noradrenaline reuptake inhibitors, and atypical antidepressants (for example, tianeptine) all increase the production of IL-10, a negative immunoregulatory cytokine and/or lower the production of IFNγ, resulting in a decreased IFNγ/IL-10 production ratio [178]. There is also evidence that SSRIs and TCAs inhibit the production of IL-1β, TNF-α, and IL-6 in brain cell cultures [65]. Also, in animal models antidepressants have antiinflammatory effects [65]. Mice challenged with a lethal dose of LPS are protected by bupropion administration, which significantly reduces the production of IFNγ, TNFα and IL-1β [179]. There is also evidence that antidepressants may attenuate inflammation-induced sickness behaviors. For example, tianeptine may reduce sickness behaviors induced by peripheral (but not central) administration of LPS and IL-1β [180]. Treatments that target inflammation, for example, etanercept that blocks TNFα functions, may attenuate IL-1β-induced sickness behaviors [181].

In depressed patients, on the other hand, the *in vivo *effects of antidepressants are less clear. Subchronic treatments with antidepressants do not consistently attenuate inflammatory signs in depressed patients [182,183]. Accordingly, a recent meta-analysis showed that antidepressant subclasses other than SSRIs did not attenuate the concentrations of pro-inflammatory cytokines [184]. Thus, despite the well-established immunoregulatory effects of antidepressants targeting inflammation (attenuate), Th1 (downregulate) and T regulatory (upregulate) functions, clinical remission in depression is not associated with normalization of immuno-inflammatory pathways [182-184]. Thus, clinical depression appears to be accompanied by a 'resistance' to the immunosuppressive effects of antidepressants [183]. This may suggest that the immuno-inflammatory pathways are continuously activated by processes that cannot be blocked by antidepressants, for example, by the autoimmune responses directed against neoantigenic determinants [133] and increased translocation of gram negative bacteria [157]. There is also evidence that antidepressants target O&NS (attenuate), antioxidants (increase) and neuroprogressive processes (attenuate) [183]. Despite these effects, increased activity and sensitization of immuno-inflammatory pathways, O&NS pathways, autoimmune responses, and neuroprogression determine in part the staging of depression, for example, treatment resistance and recurrence of depression [52]. Thus, these pathways may in part explain why in many trials the clinical efficacy of antidepressants does not outperform placebo [185] and why, despite being treated with antidepressants, depressed patients show high relapse rates [186]. Therefore, new combinatorial treatment strategies are being developed in clinical depression with drugs that target inflammation, Th1 activation, O&NS and lowered antioxidant levels, and/or neuroprogression, for example, statins, acetylsalicylic acid, minocycline, zinc, N-acelyl cysteine, curcumin, ω3 polyunsaturated fatty acids, and so on [183].

## Conclusions

**Figure 3:**
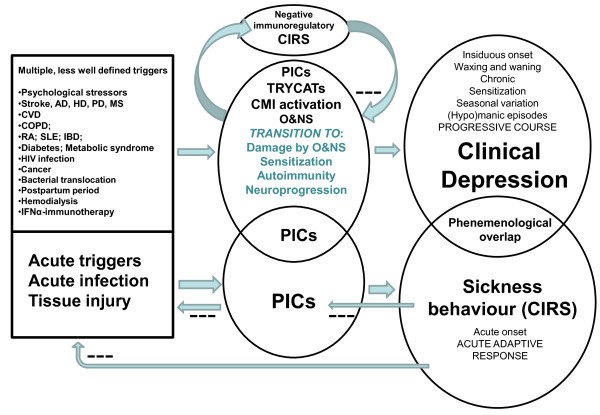
**This figure compares sickness behavior with clinical depression**. Sickness behavior is energy saving, helps to eradicate the trigger, and has anti-inflammatory effects and as such enhances recovery and is part of a compensatory (anti)-inflammatory reflex system (CIRS). Increases in pro-inflammatory cytokines (PICs) underpin both sickness behavior and clinical depression and thus may explain the partial phenomenological overlap. Depression, however, is a chronic disorder with a specific course and pathophysiology and the presence of a CIRS that downregulates the primary inflammatory response. Disorders in tryptophan catabolites (TRYCATs), cell-mediated immune (CMI) activation, and oxidative and nitrosative stress (O&NS), and, in particular, their sequelae (O&NS damage, sensitization, autoimmunity and neuroprogression) are the specific organic substrates of depression. While sickness behavior is a behavioral response to acute triggers, the onset of depression is associated with multiple less-well defined trigger factors, for example, brain disorders, such as Alzheimer (AD), Huntington (HD), and Parkinson (PD) disorder, stroke and multiple sclerosis (MS); systemic disorders, such as cardio-vascular disorder (CVD), chronic obstructive pulmonary disorder (COPD), rheumatoid arthritis (RA), systemic lupus erythematosus (SLE), inflammatory bowel disorder (IBD), diabetes, metabolic syndrome, HIV infection, cancer, bacterial translocation; and conditions, such as postpartum period, hemodialysis, and interferon-(IFN)α based immunotherapy.

Figure 3 compares sickness behavior with clinical depression. Sickness behavior helps to eradicate the trigger, has anti-inflammatory effects and is energy saving. As such sickness behavior enhances recovery and should be considered as part of a CIRS, which limits an overzealous immuno-inflammatory response to acute triggers. Cross-sectionally, there are phenomenological similarities between sickness behavior and the basic symptoms and melancholic, physio-somatic and anxiety symptom dimensions of clinical depression. Major differences, however, are that malaise, a core symptom of sickness behavior, occurs in some depressed patients only, whereas significant pyrexia is confined to sickness behavior. Moreover, depression is often accompanied not by anorexia and weight loss but by hyperphagia and weight gain. In most individuals, depression is a lifelong disease with a tendency towards recurrent episodes (waxing and waning), a chronic course, seasonal variation, and occasionally (hypo)manic symptoms. Moreover, sensitization and a potentially progressive deteriorating pattern characterize the course of depression. This contrasts with sickness behavior, which is defined as an acute, short lasting (19 to 43 days) behavioral pattern.

Translational studies show that inflammatory pathways may account for sickness behavior as well as clinical depression and, therefore, may explain the partial phenomenological overlap between both conditions. Clinical depression is accompanied by a CIRS that downregulates the primary immuno-inflammatory response and by a transition from inflammation and ROS/RNS overproduction to sensitization in inflammatory and CMI responses, progressive O&NS damage and autoimmune responses. The latter pathways appear to be the substrate of a neuroprogressive process, whereby multiple depressive episodes cause neural tissue damage and its functional and cognitive sequelae. Sickness behavior is also induced by immuno-inflammatory pathways, but in contrast to clinical depression, the sickness behavioral complex has beneficial CIRS effects enhancing recovery from the primary trigger. When there is no resolution of the acute inflammatory phase within 19 to 43 days, chronic inflammation may ensue causing transition to chronic inflammatory pathology. Sickness behavior plays a critical role in preventing this transition by compensating for the negative energy balance and redirecting energy to the activated immune cells.

Whereas acute infections typically elicit sickness behavior, there is not much evidence that acute pathogens play a major role in clinical depression. While traumatic life events can induce an inflammatory state often leading to clinical depression, no association between psychosocial stressors and sickness behavior has been described. Comorbid depression increases morbidity and mortality in patients with (auto)immune and (neuro)inflammatory disorders, whereas sickness behavior, when present, would tend to dampen the inflammatory pathways.

All in all, while sickness behavior is a beneficial CIRS response, clinical depression is a disabling, progressive disorder. Inflammation thus provokes a Janus-faced response with a good 'acute' protective inflammatory side, involving CIRS responses, such as sickness behavior, and a bad 'chronic' side, that can lead to clinical depression, a chronic disorder with positive feedback loops between (neuro)inflammation and (neuro)degenerative processes following less well-defined triggers. This Janus face also represents the transition of an inflammation-induced adaptive behavioral response that is conserved through evolution to an inflammation-related chronic progressive disorder, which is increasing in prevalence in industrialized societies [187].

## List of abbreviations

AD: Alzheimer disease; BCG: bacille Calmette-Guérin; CMI: cell-mediated immune; COPD: chronic obstructive pulmonary disease; CRP: C-reactive protein; CVD: cardiovascular disorder; DSM IV TR: The Diagnostic and Statistical Manual of Mental Disorders, fourth edition, text revision; IBD: inflammatory bowel disease; IDO: indoleamine 2,3-dioxygenase; IFN: interferon; IL-1: interleukin-1; IL-1RA: IL-1 receptor antagonist; IRS: inflammatory responses system; LPS: lipopolysaccharide; mtDNA: mitochondrial DNA; O&NS: oxidative and nitrosative stress; PD: Parkinson disease; PICs: pro-inflammatory cytokines; RA: rheumatoid arthritis; ROS/RNS: reactive oxygen and nitrogen species; sIL-2R: soluble IL-2 receptor; SLE: systemic lupus erythematosus; SSRIs: selective 5-HT reuptake inhibitors; TCAs: tricyclic antidepressants; TNF: tumor necrosis factor; TGF: transforming growth factor; Th: T helper; TRYCAT: tryptophan catabolite.

## Competing interests

There was no financial support for this specific research.

MBk has received Grant/Research Support from the NIH, Cooperative Research Centre, Simons Autism Foundation, Cancer Council of Victoria, Stanley Medical Research Foundation, MBF, NHMRC, Beyond Blue, Geelong Medical Research Foundation, Bristol Myers Squibb, Eli Lilly, Glaxo SmithKline, Organon, Novartis, Mayne Pharma and Servier, has been a speaker for Astra Zeneca, Bristol Myers Squibb, Eli Lilly, Glaxo SmithKline, Janssen Cilag, Lundbeck, Merck, Pfizer, Sanofi Synthelabo, Servier, Solvayand Wyeth, and served as a consultant to Astra Zeneca, Bristol Myers Squibb, Eli Lilly, Glaxo SmithKline, Janssen Cilag, Lundbeck and Servier. The other authors declare that they have no competing interests.

## Authors' contributions

MM and MB participated in the design of this review, while all authors helped to draft the paper. All authors contributed equally to this paper. All authors read and approved the final manuscript.

## Pre-publication history

The pre-publication history for this paper can be accessed here:

http://www.biomedcentral.com/1741-7015/10/66/prepub
